# Supplementary material for: Formation of Highly Efficient Perovskite Solar Cells by Applying Li-Doped CuSCN Hole Conductor and Interface Treatment
Source: Nanomaterials (Basel). 2022 Nov 10;12(22):3969. doi: 10.3390/nano12223969 (PMC9698157; doi:10.3390/nano12223969)
Supplement: Supplementary file 1 [file nanomaterials-12-03969-s001.zip › nanomaterials-2005370-supplementary.pdf]

Supplementary Materials

Table S1. Hole mobility and conductivity for various Li-doped CuSCN films evaluated by a Hall effect measurement system.

Li-doped CuSCNs	Mobility [$cm^2 V^{-1} s^{-1}$]	Conductivity [$\Omega^{-1} cm^{-1}$]
CuSCN	0.15	3.1×10^{-5}
Li0.18:CuSCN	0.75	1.5×10^{-4}
Li0.33:CuSCN	1.42	2.9×10^{-4}
Li0.69:CuSCN	1.02	2.1×10^{-4}

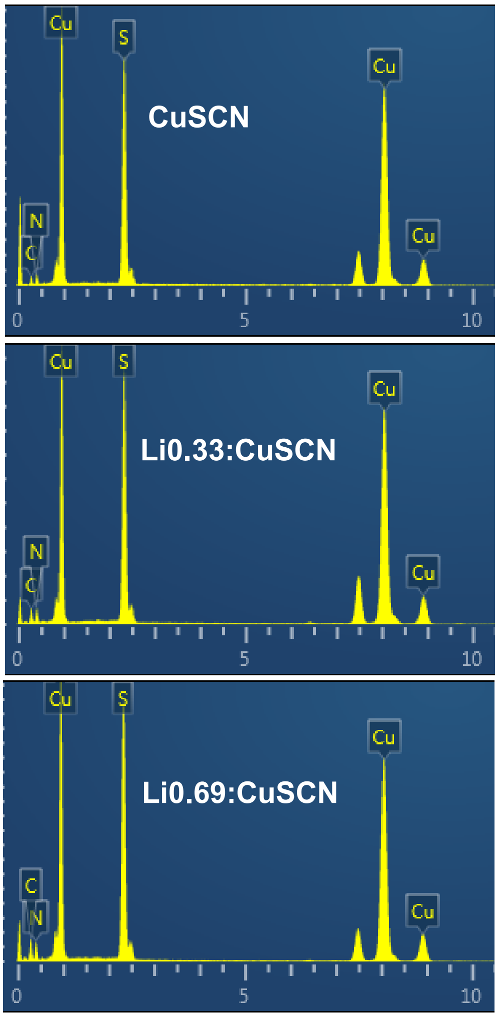

Figure S1. EDX spectra of CuSCN, Li0.33:CuSCN, Li0.69:CuSCN powders.

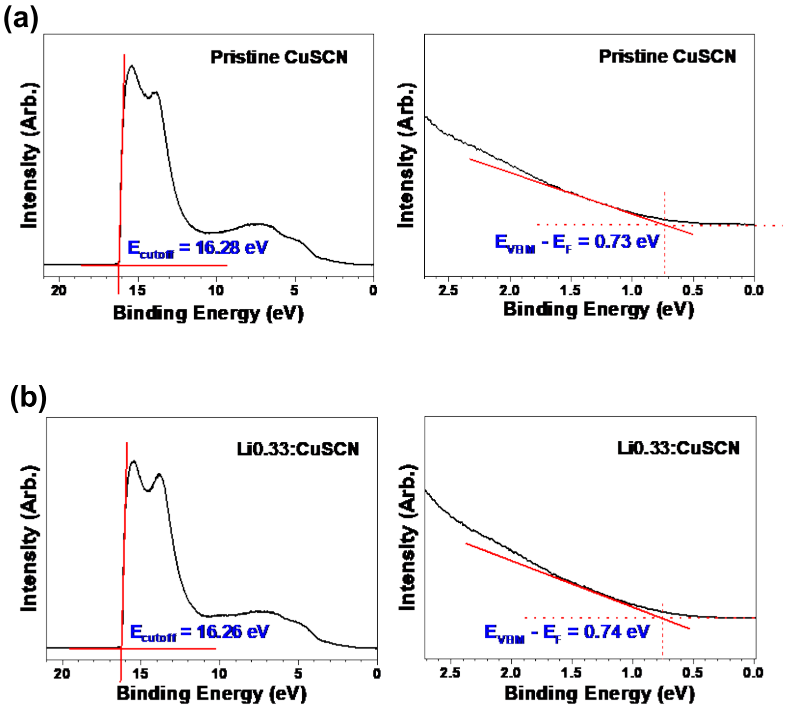

E_{VBM} = 21.22 eV - 16.26 eV + 0.74 eV = 5.70 eV

Figure S2. UPS spectra of the pristine CuSCN (a) and Li0.33:CuSCN (b) films coated on a Pyrex glass, showing their high binding-energy region (left) and low-binding energy region (right), respectively.

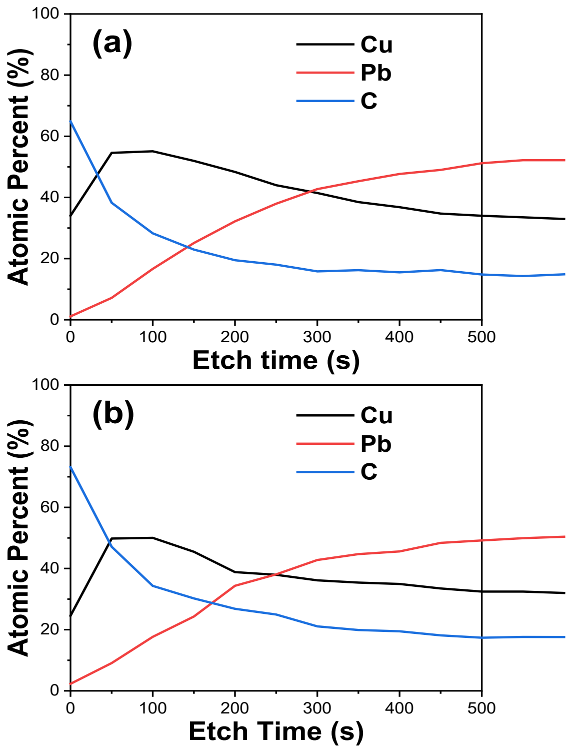

Figure S3. XPS depth profiles of Cu, Pb, and C compositions as a function of etch time for the perovskite/Li0.33CuSCN (a) and perovskite/Li0.33CuSCN/PCPDTBT (b) films.

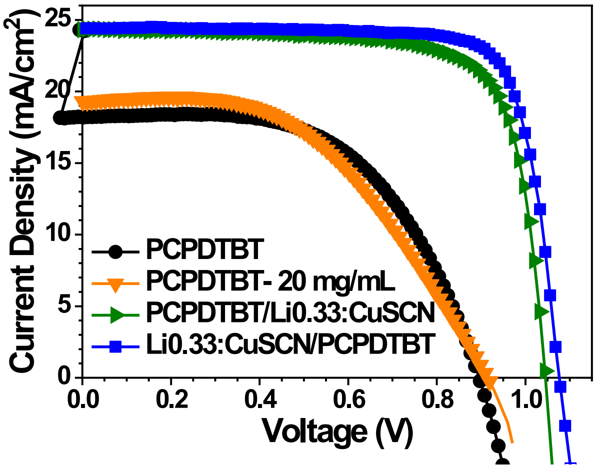

Figure S4. *J-V* curves for the PSC devices employing various HTM systems. The concentration of PCPDTBT coating solution was 0.2 mg in 1 mL chlorobenzene if its concentration was not indicated otherwise.

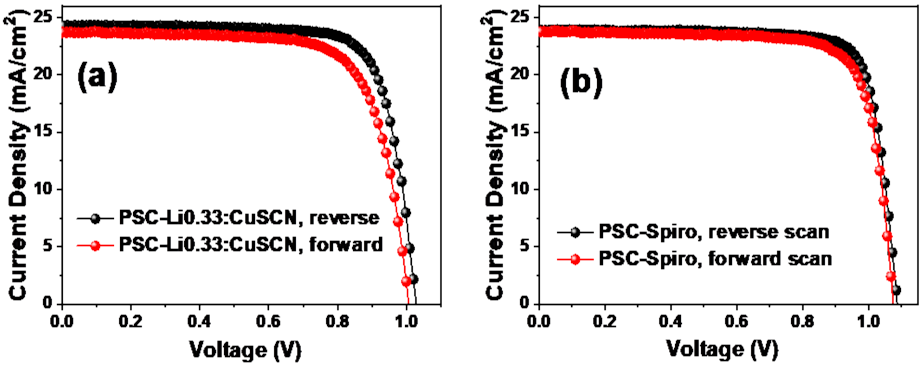

PSC-	V_{OC} (V)	J_{SC} 2	FF (%)	PCE (%)
	1.029	24.31	75.69	18.93
	1.011	23.69	72.77	17.43

PSC-Spiro	V_{OC} (V)	J_{SC} 2	FF (%)	PCE (%)
	1.089	23.93	79.08	20.61
	1.079	23.76	77.28	19.81

Figure S5. *J-V* curves for forward and reverse scans acquired from PSC-Li0.33:CuSCN (a), and PSC-spiro (b) devices.
